# Autophagy in dental tissues: a double-edged sword

**DOI:** 10.1038/cddis.2016.103

**Published:** 2016-04-14

**Authors:** H Zhuang, K Ali, S Ardu, C Tredwin, B Hu

**Affiliations:** 1 Department of Cariology, Endodontology and Operative Dentistry, Peking University School and Hospital of Stomatology, 22 South Zhongguancun Avenue, Haidian District, Beijing 100081, PR China; 2 Peninsula Dental School, Plymouth University Peninsula Schools of Medicine & Dentistry, 16 Research Way, Plymouth PL6 8BU, UK; 3 Division of Cariology & Endodontology, Dental School, University of Geneva, Geneva, Switzerland

## Tooth development and stem cells

Tooth is an essential organ in humans. Tooth development spans from the embryonic to the adolescent stage and can last for more than 10 years, which represents possibly the longest duration among all the human organs.^1^ The whole tooth development process ranges from the initial oral epithelium thickening to root formation and eruption.^1^ Early tooth development results in rapid cell proliferation to provide sufficient cell number for populating the organ. The key events during later developments are involved in the differentiation of 'tooth-specific' cells such as ameloblasts, the epithelial origin cells that produce enamel, and odontoblasts, the mesenchymal origin cells that produce dentine.^2^

The long time duration associated with the development and maturation of teeth, followed by their continuous exposure to a complex oral cavity environment, makes the teeth vulnerable to genetic, intrinsic and extrinsic influences.^3^ The consequences include failures and defects of tooth development, such as tooth agenesis,^3^ as well as being prone to trauma and diseases such as dental caries and periodontitis.

Tooth tissues have no (e.g. enamel) or very limited (e.g. dentine) regeneration capability because upon development completion only a limited number of stem cells persist in the mesenchyme (e.g. pulp and periodontal ligaments) and the epithelium disappears completely.^4^ However, increasing evidence has shown that stem cells are indeed important for tooth development and regeneration,^4^ as, besides chondrocytes and osteoblasts, tooth pulp cells have been recently shown to be able to trans-differentiate into other cell types such as neuron-like cells.^5^ Therefore, maintenance of a healthy tooth is not only important for a fully functional digestive system, but also essential to preserve an important cell source for regenerative medicine and stem cell therapies.

## Autophagy and tooth diseases

Autophagy is a major cellular process that has been implicated in a variety of cellular and tissue events, including cell stress, endogenous and exogenous cellular component clearance, development, aging and cancer.^6^ Depending on the systems, autophagy has often been linked with mitochondrial dysfunctions and autophagosomes are constantly localized inside the mitochondria.^6^ In addition, autophagy has been recently considered an important cellular event for protecting stem cells from damages by extrinsic factors.^7^ Notably, while many normal types of cells require a certain well-controlled level of autophagy, any condition beyond the capability of a cell to control can trigger a specific killing machinery: autophagic cell death.^6^

**Figure 1:**
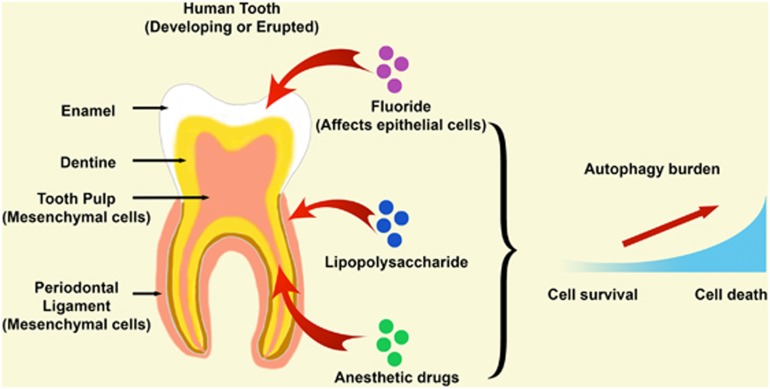
Current knowledge about autophagy induction by extrinsic factors in dental tissues. In a human tooth, autophagy has been shown to be elevated under different conditions, such as in fluorosis, in periodontal diseases (through lipopolysaccharide) and during local anesthetic treatment. The affected tooth cells can be of epithelial or mesenchymal origin depending on the specific condition and location where one factor acts (illustrated by red arrows). The role of autophagy in a tooth cell can be protective or to induce cell death, which is time and dose dependent

In the tooth, autophagy at least persists at a low level in odontoblasts and tooth pulp cells.^8, 9^ Although tooth diseases have a high prevalence, the linkage of the key cellular protection and cell death machinery (autophagy) in regard to tooth development and various dental diseases has not been researched sufficiently to date. So far, the role of autophagy in developing and adult teeth has been investigated in the context of extrinsic influences only (summarized in Figure 1).

## Drugs

Local anesthetics are widely used in dental clinics. However, the side effects of this category of drugs have been rarely studied. A recent epidemiological study showed that local anesthetics could potentially induce tooth agenesis.^10^ Using detailed dynamic cellular energetic analysis, our recent findings suggest that these drugs are able to rapidly induce autophagy in the tooth pulp cells, both in animal models and in cultured human cells.^9^ The induced autophagy is due to increased mitochondrial respiration, which is believed to counteract the toxicity of the drugs, that is, a protection mechanism.^9^ However, this protective machinery failed to function after a longer treatment at high dose, suggesting mitochondrial functions have been possibly damaged. Currently we are performing further experiments of checking mitochondria integrity and functions aiming to further improve the current drug formulations.

Besides anesthetics, other known drugs such as fluoride can also affect tooth development (known as fluorosis),^11^ primarily during differentiation. However, the precise cell protection and pathological role of autophagy has not been fully investigated so far. As functional ameloblasts and odontoblasts both bear a large amount of vacuoles that are linked to their secretory functions, we postulate that the function of drugs such as fluoride and tetracycline, on ameloblasts and odontoblasts, respectively, might be also linked with disturbed autophagy processes. Relative works are also ongoing currently in our laboratories.

## Bacteria

Microbial infection is the major factor that is involved in the etio-pathogenesis of caries and periodontitis.^12^ Lipopolysaccharide (LPS) produced by *P.* *gingivalis* has long been recognized as the key factor implicated in the initiation and development of periodontitis by activating the Toll-Like Receptor pathway.^12^ Our recent results showed that LPS can induce autophagy in the tooth periodontal ligament cells, another kind of mesenchymal cells that are key for maintaining the integrity of the periodontium, in a time-dependent manner (Zhuang *et al.*, under revision). Furthermore, the induction of autophagy has been linked with changes in cytoskeleton systems (Zhuang *et al.*, under revision). These findings highlight the protective role of autophagy in pathological processes in other types of dental diseases.

## Aging

On the other hand, in the tooth, it has also been reported that intrinsic factors, particularly aging, can result in elevated autophagy in the tooth pulp cells,^8^ suggesting potentially autophagy is also important for maintaining functional activities or survival of the differentiated odontoblasts and pulp mesenchymal cells.

## Perspectives

Based on the limited information we have till now, we understand already that autophagy has an important protective role in several tooth-related diseases and aging. Autophagy is a common mechanism in cells reacting to stress and it may not be surprising to see more studies on the expression of autophagy markers in various dental pathological conditions. However, excessive autophagy can trigger cell death. Therefore it is essential to understand the precise role and mechanisms of autophagy in distinct situations. Further researches are required to better understand the role of autophagy in the initiation and progression of different dental diseases and how autophagy is linked with the other extrinsic factors, particularly in dental caries. We believe the other challenge is to understand the linkage of autophagy to genetic factors resulting in differentiation failures leading to structural tooth defects such as amelogenesis imperfecta and dentinogenesis imperfecta, as well as conditions affecting tooth number and shape.
